# Synthesis, antimicrobial evaluation, ADMET prediction, molecular docking and dynamics studies of pyridine and thiophene moiety-containing chalcones

**DOI:** 10.1098/rsos.241411

**Published:** 2025-01-29

**Authors:** Fahmida Akhter, Sumita Saznin Marufa, S. M. Anyet Ullah Shohag, Hiroshi Nishino, Mohammad Sayed Alam, Md. Aminul Haque, Mohammad Mostafizur Rahman

**Affiliations:** ^1^ Department of Chemistry, Jagannath University, Dhaka 1100, Bangladesh; ^2^ Department of Chemical Engineering and Polymer Science, Shahjalal University of Science and Technology, Sylhet 3114, Bangladesh; ^3^ Department of Chemistry, Graduate School of Science and Technology, Kumamoto University, Kumamoto, Japan

**Keywords:** chalcone, antimicrobial activities, ADMET, docking, molecular dynamics

## Abstract

In this study, three pyridine- and four thiophene-containing chalcone derivatives were synthesized via Claisen–Schmidt condensation reaction, where five derivatives were new. Different spectral analyses (IR, ^1^H NMR, HRMS) clarified the structures and these proposed compounds were screened for antimicrobial activity by the agar disc diffusion technique. Compound **1c** was conspicuously active against most of the bacterial and fungal strains. It displayed higher activity against *Bacillus cereus* (22.3 ± 0.6 mm), *Shigella sonnei* (43.3 ± 0.6 mm) and *Shigella boydii* (34.0 ± 1.0 mm) compared to the standard ceftriaxone (20.3 ± 0.6 mm, 40.3 ± 0.6 mm and 25.7 ± 0.6 mm, respectively). In addition, the exhibited inhibition zone of compound **1c** against all fungal strains was higher than that of the standard amphotericin B. All the newly synthesized derivatives satisfied the ADME properties, and no toxicological risks were found. All compounds were docked against three protein receptors with the range of binding affinity of −6.3 to −9.6 kcal mol^−1^. Molecular dynamics simulation was scrutinized further for compound **1c** in three protein–ligand complexes where root mean square deviation and root mean square fluctuation data were below 2 Å, proposing its stability inside and minimal structural changes.

## 1. Introduction

**Figure 1.**
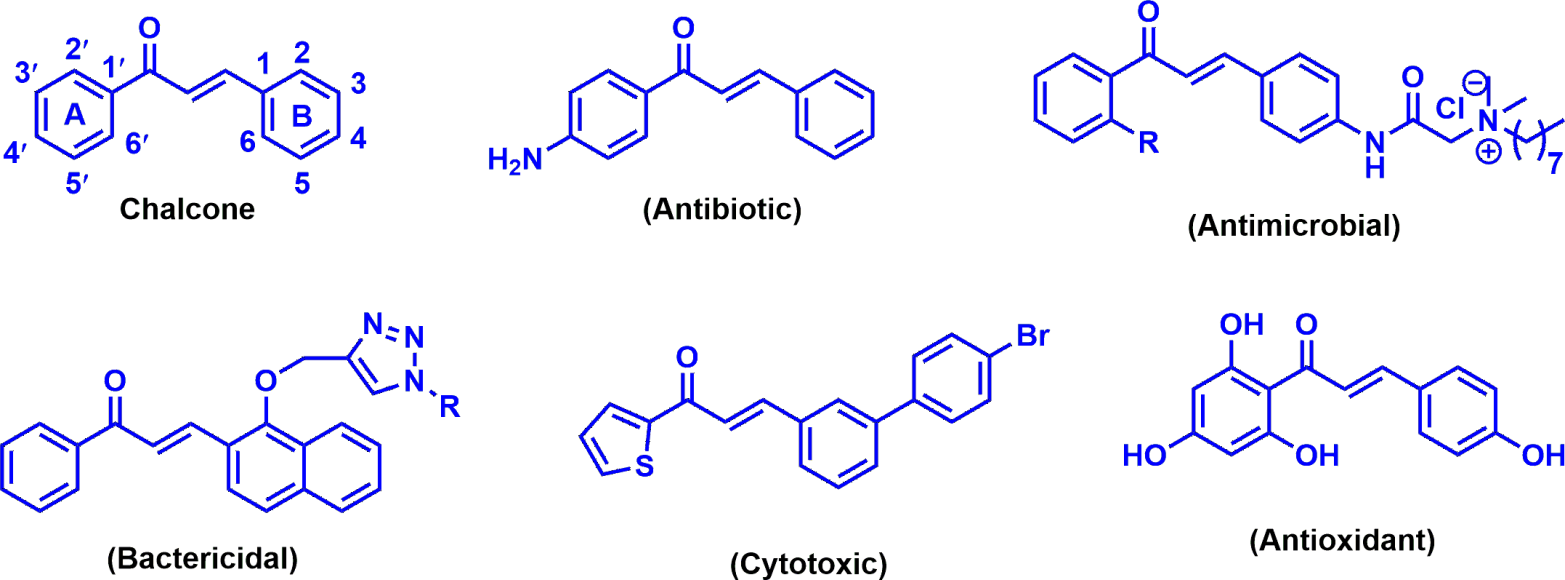
Chemical structures of chalcone and several bioactive chalcone derivatives.

**Figure 2.**
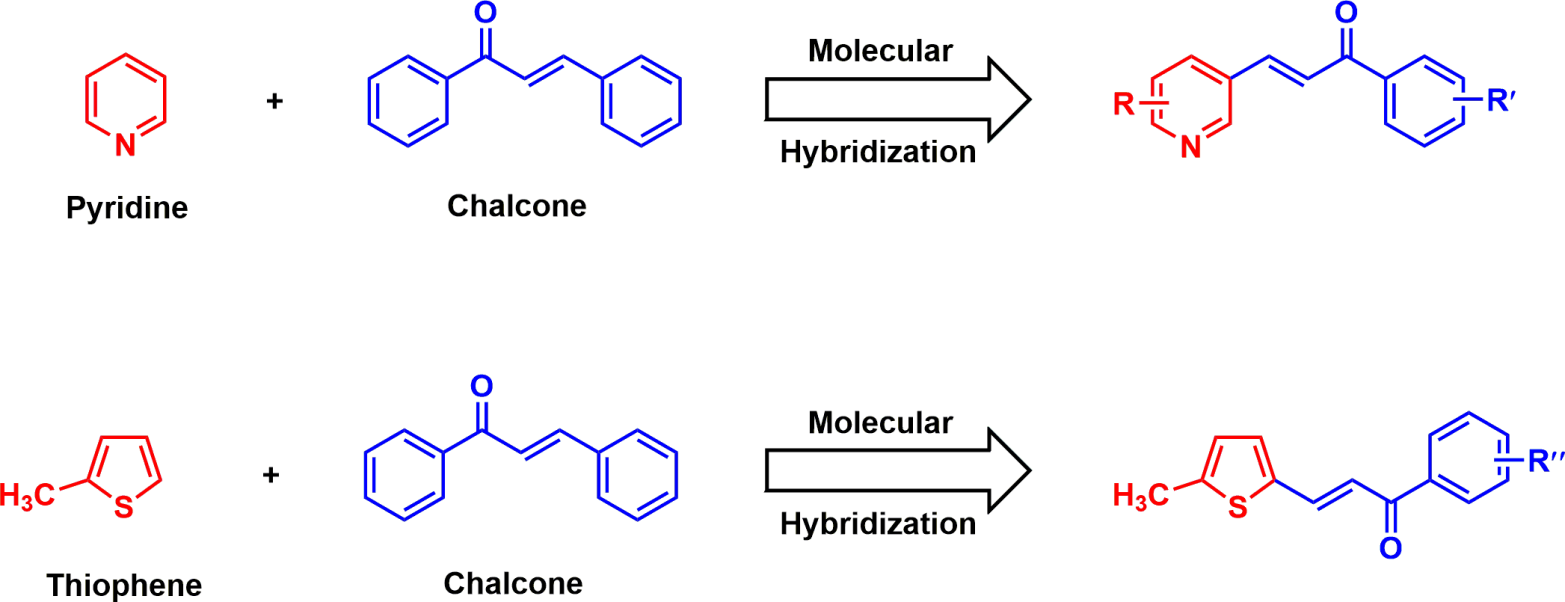
Design for the synthesis of pyridine- and thiophene-containing chalcone derivatives.

Microbial resistance to various antimicrobial agents has become a serious challenge in the healthcare sector within the last few decades around the globe. According to the survey of World Health Organization, approximately 50 000 people across the world are dying every day as a consequence of microbial infections [1]. Thus, it is a pressing need to discover and develop potential antimicrobial agents along with efficient synthesis procedures. Chemically, chalcones (1,3-diaryl-2-propen-1-ones) consist of two aromatic rings (ring A and B) containing flavonoids that are linked by α,β-unsaturated carbonyl scaffold with diverse arrangements of substituents (figure 1). This scaffold is a pioneering step for the synthesis of a variety of heterocyclic compounds. Chalcones and their derivatives secured significant attention with their multifaceted activities and simple synthesis procedure in the field of medicinal chemistry [2–4]. Numerous compounds bearing chalcone scaffold have been reported for their versatile therapeutical importance, involving antimicrobial [5,6], antioxidant [5,7], anticancer [8,9], anti-inflammatory [10,11], antitumour [12] and antimalarial [13,14] activities. Several bioactive chalcone derivatives are shown in figure 1. Biological activities of chalcones are thought to be caused by the presence of the conjugated double bond in carbonyl functionality, as the absence of this functionality renders them inert [15]. Nitrogen-containing heteroaromatic skeletons play significant roles as intermediates in the pharmaceutical field due to their diverse biological applications [16]. Pyridine is a nitrogen-containing basic heterocyclic compound having numerous therapeutic applications in medicinal science including antibacterial [17], antifungal [18], antitumour [19], anticancer [20] and antioxidant [21]. On the other hand, literature review revealed that heterocyclic thiophene derivatives are crucial scaffolds in biologically active compounds. Many thiophene derivatives are outlined with wide spectrum of bioactivity, including antibacterial [22], anti-allergic [23], anti-inflammatory [24], antioxidant [25] and anticancer [26] activities. Molecular hybridization is an effective strategy for the development of new chemical entities with advanced drug design. Integration of different pharmacophoric scaffolds from several biologically active substances could generate a new hybrid compound with enhanced activity [27,28]. On the basis of this above-mentioned scientific strategy, we herein report the design and synthesis of hybrid derivatives incorporating chalcone with pyridine and thiophene moieties (figure 2). The synthesized novel derivatives were tested for their *in vitro* antimicrobial activity using the agar disc diffusion method against some bacterial and fungal pathogens. *In silico* physicochemical and pharmacokinetic properties of the newly synthesized chemical entities were studied. Furthermore, some active compounds were proceeded for molecular docking and dynamics simulation to demonstrate the mode of interaction and stability.

## 2. Experimental

### 2.1. General methods

A Stuart™ SMP3 melting point apparatus was employed to determine the melting points of the synthesized derivatives, which are uncorrected. Infrared (IR) spectra of the specimens were recorded on a Shimadzu IRTracer-100 infrared spectrophotometer running as KBr pellets. ^1^H NMR (400 MHz) spectra of the samples were documented with a Bruker NMR spectrometer using DMSO-*d*_6_ as solvent. Chemical shift values (*δ* values) were noted in ppm relative to the internal standard TMS and coupling constant values (*J*) were measured in Hz. The spin multiplicities were represented in abbreviated form as s (singlet), d (doublet), t (triplet), q (quartet) and m (multiplet). Mass spectra were obtained using a JEOL JMS-700 MStation from the Instrumental Analysis Center, Kumamoto University, Kumamoto, Japan. All the reagents and solvents were purchased from Sigma-Aldrich and TCI Chemical Industries Ltd and used without further purification. The progress of the reactions was monitored by ascending thin layer chromatography (TLC) on silica gel F_254_ coated aluminium alloy (Merck, Germany) and visualized by exposure to UV light.

### 2.2. Synthesis

#### 2.2.1. General procedure for the preparation of 1-aryl-3-(pyridinyl)prop-2-en-1-ones (**1a**–**1c**)

**Scheme 1.**
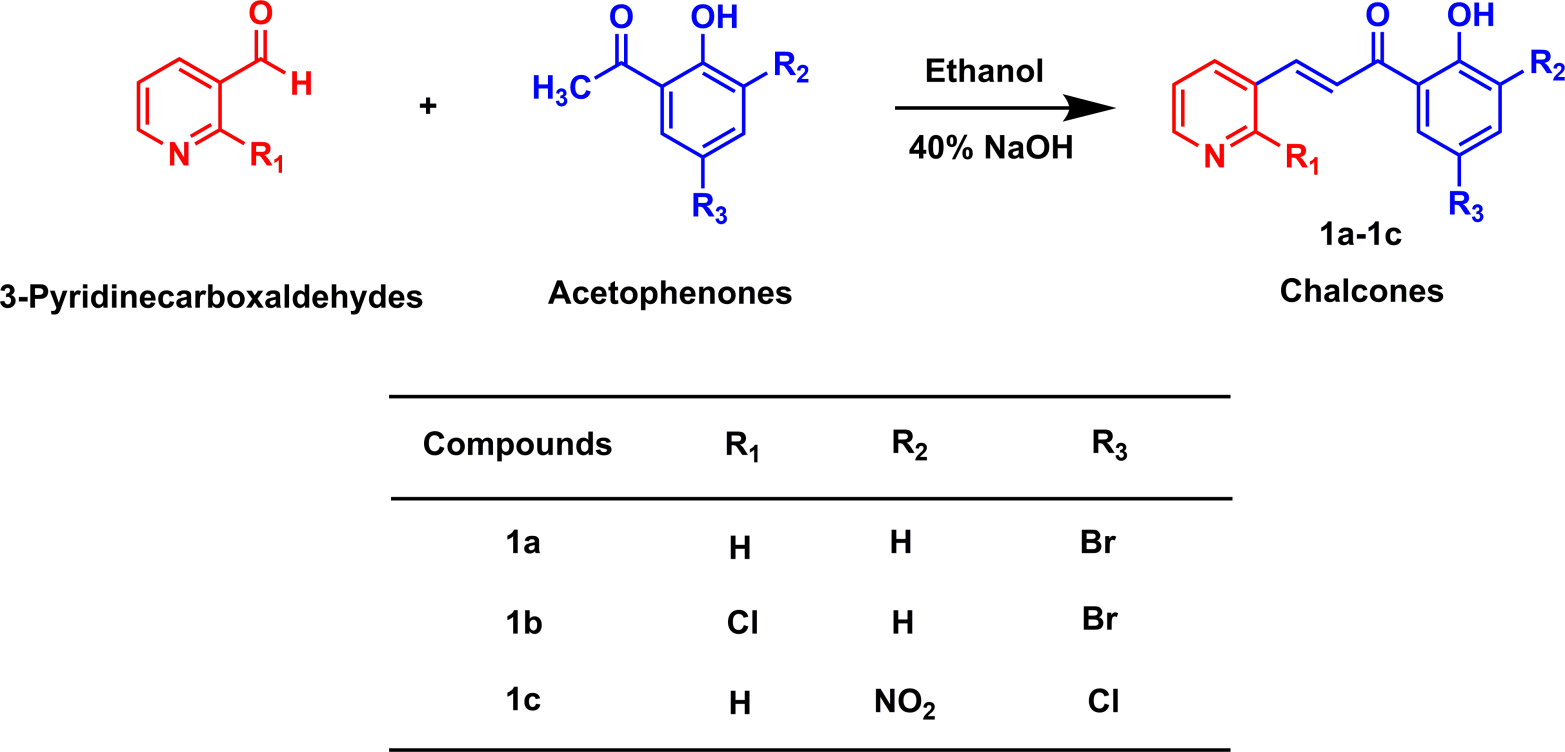
Synthesis of chalcone derivatives containing pyridine moiety.

Pyridine moiety-containing chalcone derivatives (**1a**−**1c**) were synthesized by a modified synthetic approach reported previously (Scheme 1) [29]. Briefly, an equimolar amount (0.001 mol) of 3-pyridinecarboxaldehydes and substituted acetophenones were dissolved in 10.0 ml of ethanol, coupled with 40% of aqueous NaOH solution (0.1 ml). The mixture was then stirred for a period of 3 to 4 h at room temperature until the completion of the reaction and kept overnight in a refrigerator. In the next day, the reaction mixture was acidified by 40% HCl and the mixture pot was dipped in crushed ice. The solvent diethyl ether was used to extract the aqueous layer and later the solvent was evaporated by a rotary vacuum evaporator. The extracted solid was then recrystallized from ethanol to obtain pure products **1a**−**1c**.

*1-(5-Bromo-2-hydroxyphenyl)-3-(pyridinyl)prop-2-en-1-one (**1a**)*

Yield (98%); yellow solid; m.p. 152−153°C; IR (KBr, *ʋ*_max_, cm^−1^): 3427 (O–H), 1642 (>C=O), 1578 (C=C); ^1^H NMR (400 MHz, *ẟ*, ppm, DMSO-*d*_6_): *δ* = 6.99 (d, *J* = 8.8 Hz, 1H, Ar–H), 7.51 (dd, *J* = 4.8, 3.2 Hz, 1H, Ar–H), 7.70 (dd, *J* = 6.4, 2.4 Hz, 1H, Ar–H), 7.84 (d, *J* = 16.0 Hz, 1H, =CH–C=), 8.09 (d, *J* = 16.0 Hz, 1H, =CH–CO), 8.35 (d, *J* = 2.4 Hz, 1H, Ar–H), 8.36−8.39 (m, 1H, Ar–H), 8.64 (dd, *J* = 3.2, 1.6 Hz, 1H, Ar–H), 9.04 (d, *J* = 2.0 Hz, 1H, =CH–N), 12.21 (s, 1H, O–H); FAB HRMS (acetone/NBA) calcd for C_14_H_11_BrNO_2_ 303.9973 [M+H]^+^. Found 303.9983.

*1-(5-Bromo-2-hydroxyphenyl)-3-(2-chloropyridinyl)prop-2-en-1-one (* ***1b*** *)*

Yield (96%); yellow solid; m.p. 242−243°C; IR (KBr, *ʋ*_max_, cm^−1^): 3430 (O–H), 1657 (>C=O), 1565 (C=C); ^1^H NMR (400 MHz, *ẟ*, ppm, DMSO-*d*_6_): *δ* = 6.97 (d, *J* = 8.8 Hz, 1H, Ar–H), 7.55 (dd, *J* = 4.8, 2.8 Hz, 1H, Ar–H), 7.67 (dd, *J* = 6.4, 2.8 Hz, 1H, Ar–H), 7.92 (d, *J* = 15.6 Hz, 1H, =CH–C=), 8.06 (d, *J* = 15.6 Hz, 1H, =CH–CO), 8.26 (d, *J* = 2.4 Hz, 1H, Ar–H), 8.47 (dd, *J* = 3.2, 1.6 Hz, 1H, Ar–H), 8.61 (dd, *J* = 6.0, 1.6 Hz, 1H, =CH–N), 11.98 (s, 1H, O–H); FAB HRMS (acetone/NBA) calcd for C_14_H_10_BrClNO_2_ 337.9583 [M+H]^+^. Found 337.9582.

*1-(5-Chloro-2-hydroxyphenyl)-3-(pyridinyl)prop-2-en-1-one (* ***1c*** *)*

Yield (95%); yellow solid; m.p. 150−151°C; IR (KBr, *ʋ*_max_, cm^−1^): 3422 (O–H), 1654 (>C=O), 1529 (C=C); ^1^H NMR (400 MHz, *ẟ*, ppm, DMSO-*d*_6_): 7.72 (dd, *J* = 4.8, 3.2 Hz, 1H, Ar–H), 7.91 (d, *J* = 15.6 Hz, 1H, =CH–C=), 8.11 (d, *J* = 16.0 Hz, 1H, =CH–CO), 8.36 (d, *J* = 2.4 Hz, 1H, Ar–H), 8.59 (d, *J* = 2.4 Hz, 1H, Ar–H), 8.62 (s, 1H, Ar–H), 8.75 (d, *J* = 0.8 Hz, 1H, Ar–H), 9.17 (d, *J* = 1.2 Hz, 1H, =CH–N); FAB HRMS (acetone/NBA) calcd. for C_14_H_10_ClN_2_O_4_ 305.0329 [M+H]^+^. Found 305.0324.

#### 2.2.2. General procedure for the preparation of 1-aryl-3-(5-methylthiophen-2-yl)prop-2-en-1-ones (**1d**–**1g**)

**Scheme 2.**
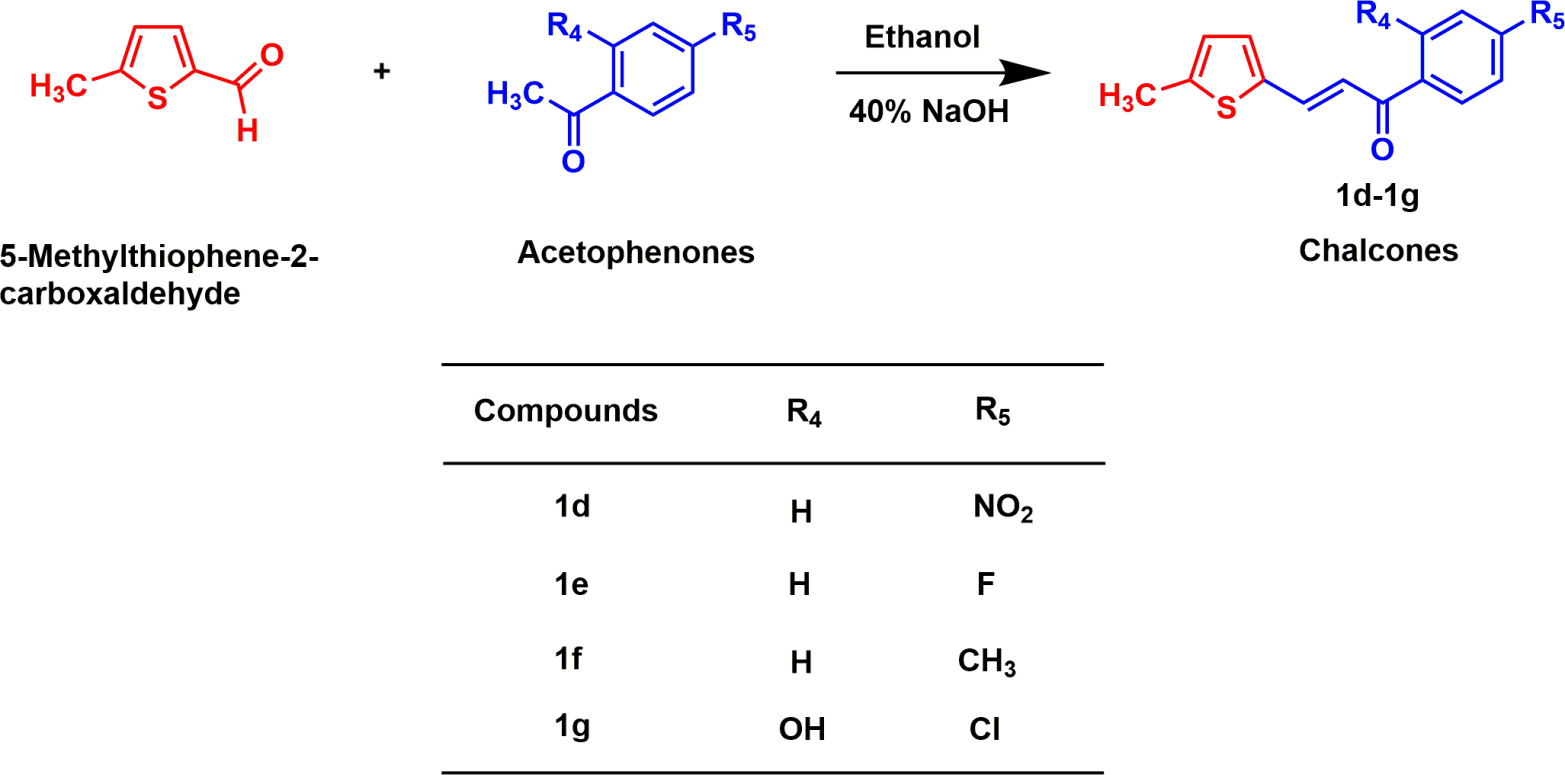
Synthesis of chalcone derivatives containing thiophene moiety.

To synthesize thiophene-containing chalcone derivatives (**1d**–**1g**), an equimolar amount (0.001 mol) of 5-methylthiophen-2-carboxaldehyde and substituted acetophenones were dissolved in ethanol (10.0 ml) and 40% of aqueous NaOH solution (0.1 ml) (Scheme 2). The mixture was then stirred for a period of 3 to 6 h at room temperature until the completion of the reaction. The reaction mixture was kept overnight in a refrigerator. The following day, 40% HCl solution was used to acidify the reaction mixture and the mixture container was kept in an ice bath. The aqueous layer was extracted with diethyl ether and the solvent was evaporated by a rotary vacuum evaporator. The solid was then recrystallized from ethanol to yield pure products **1d**−**1g**.

*3-(5-Methylthiophen-2-yl)-1-(4-nitrophenyl)prop-2-en-1-one (* ***1d*** *)*

Yield (85%); yellow solid; m.p. 187−188°C; IR (KBr, *ʋ*_max_, cm^−1^): 1650 (>C=O), 1574 (C=C), 703 (C–S–C); ^1^H NMR (400 MHz, *ẟ*, ppm, DMSO-*d*_6_): *δ* = 2.53 (s, 3H, CH_3_), 6.94 (d, *J* = 3.6 Hz, 1H, Ar–H), 7.40 (d, *J* = 15.2 Hz, 1H, =CH–C=), 7.55 (d, *J* = 3.6 Hz, 1H, Ar–H), 7.90 (d, *J* = 15.2 Hz, 1H, =CH–CO), 8.28 (d, *J* = 8.8 Hz, 2H, Ar–H), 8.36 (d, *J* = 8.8 Hz, 2H, Ar–H); FAB HRMS (acetone/NBA) calcd for C_14_H_12_NO_3_S 274.0538 [M+H]^+^. Found 274.0561.

*1-(4-Fluorophenyl)-3-(5-methylthiophen-2-yl)prop-2-en-1-one (* ***1*** ***e**)*

Yield (65%); brown solid; m.p. 74−75°C; IR (KBr, *ʋ*_max_, cm^−1^): 1658 (>C=O), 1601, 1586 (C=C), 792 (C–S–C); ^1^H NMR (400 MHz, *ẟ*, ppm, DMSO-*d*_6_): *δ* = 2.51 (s, 3H, CH_3_), 6.91 (d, *J* = 3.2 Hz, 1H, Ar–H), 7.23−7.43 (m, 3H, Ar–H, =CH–C=), 7.51 (d, *J* = 3.6 Hz, 1H, Ar–H), 7.83 (d, *J* = 15.2 Hz, 1H, =CH–CO), 8.17 (dd, *J* = 5.6, 3.2 Hz, 2H, Ar–H); FAB HRMS (acetone/NBA) calcd for C_14_H_12_FOS 247.0593 [M+H]^+^. Found 247.0604.

*1-(4-Methylphenyl)-3-(5-methylthiophen-2-yl)prop-2-en-1-one (* ***1f*** *)*

Yield (60%); yellow solid; m.p. 72−73°C; IR (KBr, *ʋ*_max_, cm^−1^): 1656 (>C=O), 1608, 1587 (C=C), 801 (C–S–C); ^1^H NMR (400 MHz, *ẟ*, ppm, DMSO-*d*_6_): *δ* = 2.39 (s, 3H, phenyl CH_3_), 2.49 (s, 3H, thiophene CH_3_), 6.90 (d, *J* = 2.4 Hz, 1H, Ar–H), 7.34−7.40 (m, 3H, Ar–H, =CH–C=), 7.48 (d, *J* = 3.2 Hz, 1H, Ar–H), 7.80 (d, *J* = 15.2 Hz, 1H, =CH–CO), 7.96 (d, *J* = 8.0 Hz, 2H, Ar–H); FAB HRMS (acetone/NBA) calcd for C_15_H_15_OS 243.0844 [M+H]^+^. Found 243.0854.

*1-(4-Chloro-2-hydroxyphenyl)-3-(5-methylthiophen-2-yl)prop-2-en-1-one (* ***1g*** *)*

Yield (70%); brown solid; m.p. 123−124°C; IR (KBr, *ʋ*_max_, cm^−1^): 3424 (O–H), 1601 (C=O stretching), 1567 (C=C), 794 (C–S–C); ^1^H NMR (400 MHz, *ẟ*, ppm, DMSO-*d_6_*): *δ* = 2.49 (s, 3H, CH_3_), 6.93 (d, *J* = 2.8 Hz, 1H, Ar–H), 7.02 (d, *J* = 3.2 Hz, 1H, Ar–H), 7.07 (s, 1H, Ar–H), 7.45 (d, *J* = 15.2 Hz, 1H, =CH–C=), 7.53 (d, *J* = 3.2 Hz, 1H, Ar–H), 7.90 (d, *J* = 15.2 Hz, 1H, =CH–CO), 8.07 (d, *J* = 8.4 Hz, 1H, Ar–H), 12.56 (s, 1H, OH); FAB HRMS (acetone/NBA) calcd. for C_14_H_12_ClO_2_S 279.0247 [M+H]^+^. Found 279.0293.

### 2.3. *In vitro* antimicrobial screening

*In vitro* antimicrobial screening of the new derivatives was carried out against several bacterial and fungal strains by the agar disc diffusion method [30,31]. As testing media, Mueller Hinton Agar (for bacteria) and Potato Dextrose Agar (for fungus) were prepared for the evaluation of the antimicrobial activities. The broth medium was incubated for a period of 24 h after it set well in a Petri dish. Following checked the contamination, the test organism was injected on the agar surface in an even fashion by swabbing the sterilized cotton bar. After the surface got dry, discs were placed gently on the agar plates. Each disc was charged with 50 µl of sample solution dissolved in DMSO and the concentration was 300 µg per disc. Standard ceftriaxone and amphotericin B in DMSO were injected on separate discs (50 µl) for antibacterial and antifungal assays, respectively, as positive control and the concentration was 50 µg per disc. Injected plates were incubated then for 24 h at 37°C for antibacterial assay and 48 h at 26°C for antifungal assay. The zones of inhibition produced by the synthesized compounds and the standards were measured in millimetres for comparison. The zone of inhibition was shown as mean value of three repeated experiments. Three Gram-positive bacterial strains, namely *Staphylococcus aureus* (*S. aureus*), *Bacillus cereus* (*B. cereus*) and *Bacillus megaterium* (*B. megaterium*), and eight Gram-negative bacterial strains, namely enterotoxigenic *Escherichia coli* (*ETEC*), enteropathogenic *Escherichia coli* (*EPEC*), *Salmonella typhi* (*S. typhi*), *Shigella flexneri* (*S. flexneri*), *Shigella sonnei* (*S. sonnei*), *S. boydii*, *Escherichia coli* (*E. coli*) and *Shigella dysentery* (*S. dysentery*), were used for antibacterial assays. Six fungal strains, namely *Aspergillus niger* (*A. niger*), *Candida albicans* (*C. albicans*), *Trichoderma harzianum* (*T. harzianum*), *Neurospora crassa* (*N. crassa*), *Penicillium notatum* (*P. notatum*) and *Aspergillus flavus* (*A. flavus*), were used for antifungal assays.

### 2.4. *In silico* toxicity, drug-likeness, and drug-score properties

ADME (absorption, distribution, metabolism and excretion) properties were considered to calculate using the SwissADME online server and the percentage of absorption (%ABS) was obtained from the following equation

(2.1) %ABS=109−(0.3459×TPSA),

where TPSA is topological polar surface area.

Additionally, Osiris Property Explorer (https://www.organic-chemistry.org/prog/peo) was operated to predict the overall toxicities, drug-likeness and drug-score of the compounds and standards on the basis of a previous report [32].

### 2.5. Molecular docking study

According to wet laboratory performance, three crystal structures of target proteins, (i) tyrosinase from *B. megaterium*, PDB ID: 3NM8 (resolution 2.0 Å) [33], (ii) periplasmic protein from *S. dysentery*, PDB ID: 2R7A (resolution 2.05 Å) [34], and (iii) beta-glucosidase from *T. harzianum*, PBD ID: 5JBO (resolution 1.97 Å) [35], were chosen and retrieved from the RCSB protein data bank for molecular docking purposes. The crystal structure of target proteins was cleaned up initially by eliminating all the heteroatoms, water molecules and inhibitors that were present in the structure using PyMol software tool (version 2.4). Swiss-PDB viewer (version 4.1.0) was involved in energy minimization and verifying the proteins’ crystal structures depending on the least amount of energy. The Gaussian 09 software suite was utilized to optimize all synthesized chalcones by the DFT method with a basis set of B3LYP/6-31G in a gaseous state. Molecular docking was accomplished by AutoDock Vina using the PyRx interface targeting the whole protein. The ligand–protein binding affinities were assessed in kcal mol^−1^ units. Binding stance and non-bonding touch were depicted via BIOVIA Discovery Studio 4.5.

### 2.6. Molecular dynamics simulation

Molecular dynamics (MD) simulations were performed using YASARA dynamics program version 21.6.17 on the three selected **1c**–3NM8, **1c**–2R7A and **1c**–5JBO complexes due to their excellent molecular docking score as well as their antimicrobial screening outcome. Simulations were run to ascertain the impact of the biological system on the protein and docked complexes. AMBER14 served as a force field to illustrate the macromolecular system. Water and Na^+^/Cl^−^ ions (0.9%) were added to neutralize the system. MD simulations were run using the particle-mesh Ewald (PME) method for computing long-range electrostatic interactions at a cut-off distance of 8 Å. Throughout the simulation period, the physiological conditions were maintained as 298 K temperature, pH 7.4 and 0.9% of NaCl with a constant pressure of 1 atm. Finally, a simulation was run for 30 ns saving snapshots at every 100 ps. From the MD simulations, some computed data like root mean square deviation (RMSD) and root mean square fluctuation (RMSF) were collected and analysed.

## 3. Results and discussion

### 3.1. Chemistry

In this study, two series of chalcones bearing pyridine and thiophene moieties were synthesized using the Claisen–Schmidt condensation reaction with high yields (60–98%). The first series (**1a**−**1c**) were obtained by the condensation of 3-pyridinecarboxaldehyde and substituted acetophenones (Scheme 1) and the second series (**1d**−**1g**) were obtained from the condensation products of 5-methylthiophene-2-carboxaldehyde and substituted acetophenones (Scheme 2). The synthesis of compound **1a** was reported to study its structure by PMR method [36]. On the other hand, the synthesis of compound **1d** was reported via a palladium-catalysed Heck-type coupling reaction [37]. Different spectroscopic techniques were operated to confirm the structures of the synthesized derivatives. For instance, the IR spectrum of **1a** exhibited absorption bands at 3427, 1642 and 1578 cm^−1^ for OH stretching, >C=O stretching, and C=C stretching, respectively [38,39]. The ^1^H NMR spectrum of **1a** displayed a single proton doublet at 6.99 ppm and two one-proton doublet doublets at 7.51 ppm and 7.70 ppm, respectively, for aromatic protons. Other four aromatic protons (one proton each) appeared at 8.35 ppm, 8.36−8.39 ppm, 8.64 ppm and 9.04 ppm as doublet, multiplet, doublet of doublet and doublet, respectively. Two single-proton doublets at 7.84 ppm and 8.09 ppm were observed for =CH–C= and =CH–CO protons, respectively. The OH proton appeared at 12.21 ppm as a singlet. Respective structures were in good agreement with the calculated mass spectrum data.

### 3.2. *In vitro* antimicrobial screening

The antimicrobial susceptibility of the synthesized analogues and standards were assessed using the agar disc diffusion method against three Gram-positive bacteria, nine Gram-negative bacteria, and six fungal strains. Tables 1–3 present the inhibition scores of the tested compounds and standards, which were determined by measuring the diameter of the inhibition zones in millimetres. The synthesized compounds and standards were evaluated at a concentration of 300 μg per disc and 50 μg per disc, respectively. Among the evaluated derivatives, compound **1c** exhibited excellent activity with a greater zone of inhibition (22.3 ± 0.6 mm) than standard ceftriaxone (20.3 ± 0.6 mm) against *B. cereus* Gram-positive bacterial strain. Compound **1c** contains electron-withdrawing nitro and chloro groups that might promote the higher activity of the compound against most of the bacterial and fungal strains which was reported in a previous study as well [40]. This compound was also moderately active against the other two Gram-positive bacterial strains. Additionally, compounds **1a** and **1g** showed adequate antibacterial activity against *B. cereus* and *B. megaterium* bacterial strains compared to standard ceftriaxone (table 1). In the case of Gram-negative bacterial strains, compounds **1a** and **1c** showed higher activities against *S. sonnei* with zones of inhibition 40.7 ± 0.6 mm and 43.3 ± 0.6 mm than standard ceftriaxone (40.3 ± 0.6 mm). Compound **1c** also showed greater activity (34.0 ± 1.0 mm) against *S. boydii* bacterial strain compared to standard ceftriaxone (25.7 ± 0.6 mm). Other compounds have considerably low or no activity against other Gram-negative bacterial strains (table 2). On the other hand, most of the compounds exhibited significant antifungal activities in terms of zones of inhibition value than the standard amphotericin B. Compound **1a** produced higher zones of inhibition against all the tested fungal strains except *T. harzianum* and *N. crassa*, although it showed moderate activity against those two strains. Compound **1b** was significantly active against all fungal strains except *T. harzianum*. Compound **1c** displayed exceptionally good activities against all strains in comparison to the standard. Compound **1d** generated comparable zones of inhibition compared to standard amphotericin B against four tested fungal strains, whereas it was not active against *A. niger* and *N. crassa* fungal strains. The presence of electron-withdrawing nitro group might be responsible for the higher activity of compound **1d** against various fungal strains which was supported by previous report [41]. Compounds **1e** and **1f** showed remarkable activities against most of the fungal strains. However, compound **1g** exhibited reduced activities compared to other synthesized derivatives against all fungal strains (table 3).

**Table 1.** Diameter of inhibition zones in mm of the synthesized chalcone derivatives **1a**–**1g** and ceftriaxone (Cef.) against different Gram (+) bacterial strains. The data are expressed as mean ± s.d. (standard deviation) of three experiments. — Represents no activity.

compound	Gram (+) bacteria		
	*S. aureus*	*B. cereus*	*B. megaterium*
**1a**	15.0 ± 1.0	17.0 ± 1.0	23.0 ± 1.0
**1b**	15.0 ± 1.0	8.0 ± 1.0	10.0 ± 1.0
**1c**	33.0 ± 1.0	22.3 ± 0.6	35.3 ± 0.6
**1d**	13.3 ± 0.6	7.3 ± 0.6	12.3 ± 0.6
**1e**	10 .0 ± 1.0	7.7 ± 0.6	7.0 ± 1.0
**1f**	11.0 ± 1.0	7.0 ± 1.0	12.0 ± 1.0
**1g**	12.7 ± 0.6	15.3 ± 0.6	14.7 ± 0.6
Cef.	40.3 ± 0.6	20.3 ± 0.6	50.7 ± 1.15
DMSO	—	—	—

**Table 2.** Diameter of inhibition zones in mm of the synthesized chalcone derivatives **1a**–**1g** and ceftriaxone (Cef.) against different Gram (−) bacterial strains. The data are expressed as mean ± s.d. of three experiments. — Represents no activity.

compound	Gram (−) bacteria							
	*ETEC*	*EPEC*	*S. typhi*	*S. flexneri*	*S. sonnei*	*S. boydii*	*E. coli*	*S. dysentery*
**1a**	13.7 ± 0.6	15.0 ± 1.0	18.7 ± 0.6	17.0 ± 1.0	40.7 ± 0.6	15.3 ± 0.6	15.0 ± 1.0	18.7 ± 0.6
**1b**	15.7 ± 0.6	9.0 ± 1.0	5.0 ± 1.0	10.0 ± 1.0	12.3 ± 0.6	14.7 ± 0.6	13.3 ± 0.6	10.0 ± 1.0
**1c**	15.3 ± 0.6	23.3 ± 0.6	15.0 ± 1.0	26.3 ± 0.6	43.3 ± 0.6	34.0 ± 1.0	10.0 ± 1.0	38.0 ± 1.0
**1d**	11.7 ± 0.6	11.3 ± 0.6	15.3 ± 0.6	6.0 ± 1.0	10.3 ± 0.6	—	11.0 ± 1.0	7.3 ± 0.6
**1e**	12.0 ± 1.0	6.0 ± 1.0	10.7 ± 0.6	6.7 ± 0.6	7.7 ± 0.6	10.0 ± 1.0	6.3 ± 0.6	6.7 ± 0.6
**1f**	15.0 ± 1.0	10.7 ± 0.6	8.7 ± 0.6	6.3 ± 0.6	8.0 ± 1.0	10.0 ± 1.0	10.7 ± 0.6	7.7 ± 0.6
**1g**	17.0 ± 1.0	13.7 ± 0.6	12.7 ± 0.6	16.3 ± 0.6	14.3 ± 0.6	12.7 ± 0.6	7.3 ± 0.6	6.3 ± 0.6
Cef.	45.3 ± 0.6	46.0 ± 0.3	44.3 ± 0.6	39.3 ± 0.6	40.3 ± 0.6	25.7 ± 0.6	38.3 ± 0.6	47.7 ± 0.6
DMSO	—	—	—	—	—	—	—	—

**Table 3.** Diameter of inhibition zones in mm of the synthesized chalcone derivatives **1a**–**1g** and amphotericin B (Amp. B) against different fungal strains. The data are expressed as mean ± s.d. of three experiments. — Represents no activity.

compound	fungi					
	*A. niger*	*C. albicans*	*T. harzianum*	*N. crassa*	*P. notatum*	*A. flavus*
**1a**	12.0 ± 1.0	17.0 ± 1.0	14.0 ± 1.0	12.3 ± 0.6	30.3 ± 0.6	16.7 ± 0.6
**1b**	16.3 ± 0.6	14.3 ± 0.6	—	15.0 ± 1.0	28.7 ± 0.6	16.0 ± 1.0
**1c**	18.3 ± 0.6	18.3 ± 0.6	20.0 ± 1.0	15.7 ± 1.0	18.7 ± 0.6	17.0 ± 1.0
**1d**	—	20.7 ± 0.6	15.0 ± 1.0	—	15.3 ± 0.6	12.3 ± 0.6
**1e**	8.7 ± 0.6	21.0 ± 1.0	16.3 ± 0.6	13.0 ± 1.0	15.0 ± 1.0	12.3 ± 0.6
**1f**	6.0 ± 1.0	16.0 ± 1.0	15.3 ± 0.6	15.3 ± 0.6	16.7 ± 0.6	14.7 ± 0.6
**1g**	7.3 ± 0.6	12.3 ± 0.6	6.7 ± 0.6	13.7 ± 0.6	8.0 ± 1.0	6.0 ± 1.0
Amp. B	8.3 ± 0.6	16.7 ± 0.6	17.7 ± 0.6	15.3 ± 0.6	13.0 ± 1.0	11.3 ± 0.6
DMSO	—	—	—	—	—	—

### 3.3. *In silico* toxicity, drug-likeness and drug-score properties

**Figure 3.**
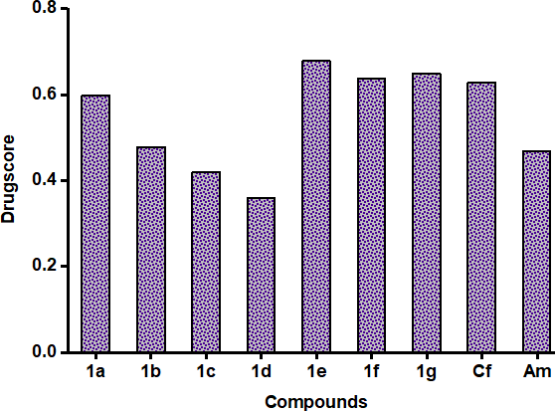
Predicted drug-score of synthesized compounds **1a**–**1g**, ceftriaxone (Cf), and amphotericin B (Am).

Physicochemical and pharmacokinetic properties are crucial computational assessments in the early stages of drug discovery and development. These predictions are derived from a compound’s molecular structure and known toxicological data to eliminate harmful candidates before conducting any expensive *in vitro* and *in vivo* testing. Lipinski’s Rule of Five and Veber’s Rule are widely used to evaluate the pharmacokinetic properties of compounds. These rules consider various physiochemical standards like molecular weight (MW), number of hydrogen bond acceptors (HBA), number of hydrogen bond donors (HBD), number of rotatable bonds (NROTB), lipophilicity (clogP), topological polar surface area (TPSA), solubility parameter (logS) and percentage of absorption (%ABS). Pharmacokinetic properties and oral bioavailability of a drug candidate are closely related and often influence each other. TPSA is used to predict a compound’s ability to permeate cell membranes. It is crucial in drug design and is linked to a drug’s oral bioavailability. A TPSA of 140 Å² or less is typically preferred for good oral bioavailability. All our synthesized compounds showed a TPSA value between 45.31 Å² and 96.01 Å² (table 4). The solubility values (logS) of our synthesized compounds ranged from −3.58 to −4.76, falling within the standard range (0.5 to −6.5) [42]. Table 4 provides an overview of ADME predictions for synthetic derivatives. All the compounds agreed with Lipinski’s Rule of Five and Veber’s Rule, which strongly favour their oral bioavailability. Compound **1c** was inferred to have favourable lipophilicity over the other compounds owing to the lower clogP value (1.64) [43]. To predict the adverse effect of the analogues, the toxicological parameter (mutagenic, tumorigenic, irritant and reproductive), drug-likeness, and the drug score were considered and presented in table 5. The main cause of toxicity risk in a substance is the presence of certain groups. According to reports, most medications were unable to pass clinical trials due to significant risk factors for toxicity. In this investigation, all the tested compounds displayed low mutagenic, tumorigenic, irritant and reproductive effects. The predicted drug score of the synthesized compounds **1a**−**1g** ranged from 0.36 to 0.68 as shown in figure 3. Compound **1e** showed the highest drug-likeness of 3.35 and a drug score of 0.68 compared to he others. *In silico* ADME, toxicity risk, drug-likeness and drug-score analyses indicated that these compounds could become effective drugs in the future.

**Table 4.** Pharmacokinetic properties of compounds **1a**–**1g**, ceftriaxone (Cef.), and amphotericin B (Amp. B).

compound	Lipinski’s violations	Lipinski’s rule				Veber’s rule		logS^g^	%ABS^h^
		MW^a^ (≤500)	HBA^b^ (≤10)	HBD^c^ (≤5)	clogP^d^ (≤5)	NROTB^e^ (≤10)	TPSA^f^ (140 Å^2^)		
**1a**	0	303	3	1	2.68	3	50.19	−3.58	91.64
**1b**	0	337	3	1	3.39	3	50.19	−4.08	91.64
**1c**	0	305	5	1	1.64	4	96.01	−3.94	75.79
**1d**	0	273	3	0	2.67	4	91.13	−4.76	77.48
**1e**	0	246	2	0	3.70	3	45.31	−4.62	93.33
**1f**	0	242	1	0	3.94	3	45.31	−4.65	93.33
**1g**	0	278	2	1	3.86	3	66.54	−4.74	85.98
Cef.	1	331	5	1	−1.53	3	72.88	−3.32	83.85
Amp. B	1	416	3	0	4.85	6	27.05	−5.08	99.67

^a^ Molecular weight.

^b^ Number of hydrogen bond acceptors.

^c^ Number of hydrogen bond donors.

^d^ Lipophilicity.

^e^ Number of rotatable bonds.

^f^ Topological polar surface area.

^g^ Solubility parameter.

^h^ Percentage of absorption.

**Table 5.** *In silico* toxicity risks, drug-likeness and drug score of compounds **1a**–**1g**, ceftriaxone (Cef.), and amphotericin B (Amp. B). Toxicity effects are shown as M, mutagenic; T, tumorigenic; I, irritant; R, reproductive.

compound	M	T	I	R	drug-likeness
**1a**	low	low	low	low	−0.18
**1b**	low	low	low	low	−0.82
**1c**	low	low	low	low	−4.02
**1d**	low	low	low	low	−8.98
**1e**	low	low	low	low	3.35
**1f**	low	low	low	low	2.16
**1g**	low	low	low	low	3.14
Cef.	low	low	low	low	16.69
Amp. B	low	low	low	low	0.14

#### 3.3.1. Molecular docking study

**Figure 4.**
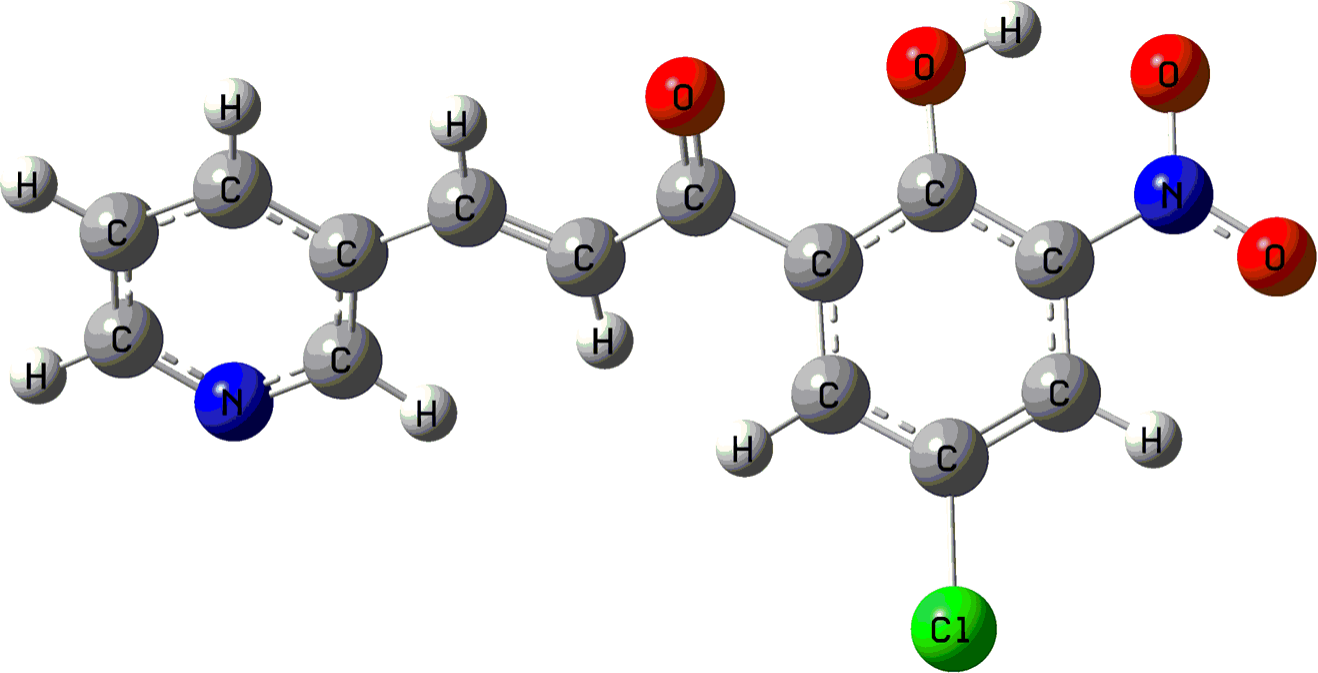
Optimized molecular structure of synthesized compound **1c** with a basis setup B3LYP/6-31G.

**Figure 5.**
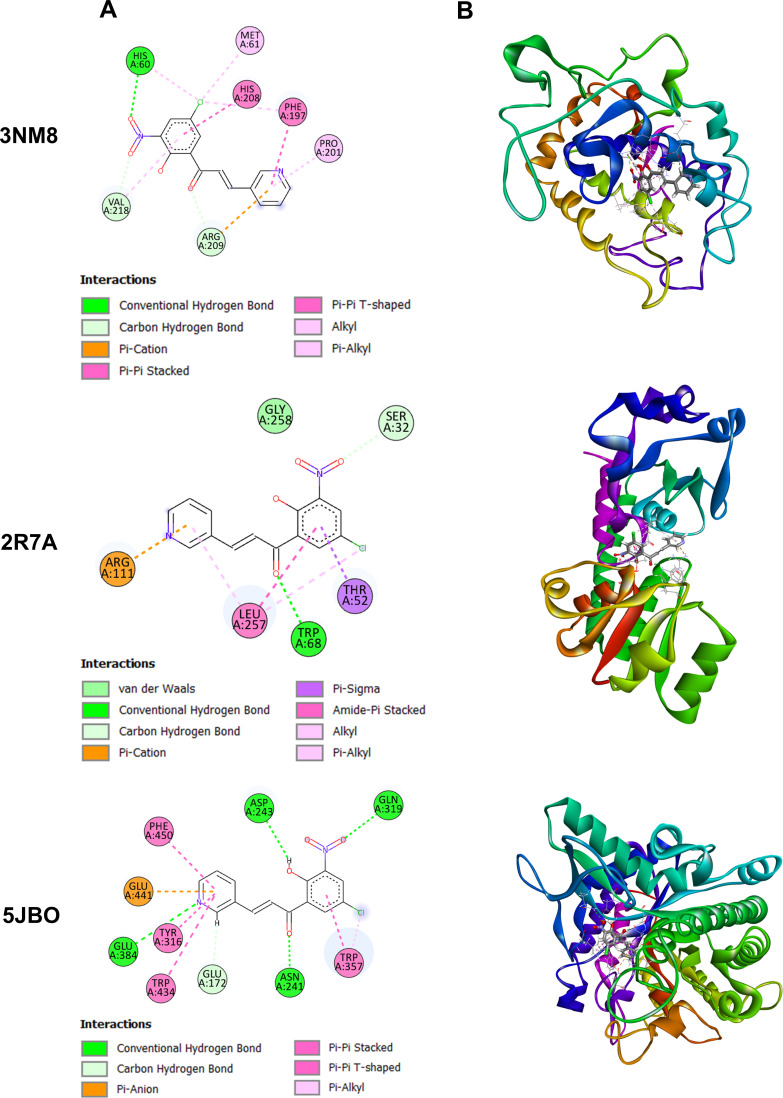
Two-dimensional interaction profile (*a*) and three-dimensional docking simulation (*b*) of **1c** against 3NM8, 2R7A, and 5JBO protein receptors.

Molecular docking was carried out via AutoDock Vina interphase of PyRx 0.8, against three selective protein receptors namely tyrosinase enzymes of *B. megaterium* (PDB ID: 3NM8), periplasmic protein of *S. dysentery* (PDB ID: 2R7A) and β-glucosidase enzymes of *T. harzianum* (PDB ID: 5JBO). The DFT/B3LYP/6-31G technique was used for the optimization process. The optimized molecular structure of **1c** is depicted in figure 4. Nonbonding interactions were analysed using PyMOL 2.4 and Discovery Studio 4.5 software. Table 6 illustrates the binding affinity of all the compounds. Compound **1c** showed maximum binding scores against all protein receptors. The two-dimensional and three-dimensional interaction profiles of **1c** against the three protein receptors are shown in figure 5. When compound **1c** was docked against 3NM8, it displayed a binding affinity value of −7.4 kcal mol^−1^. Arg209 residue of tyrosinase enzymes generated one carbon–hydrogen bond with the carbonyl oxygen and one electrostatic pi-cation interaction with the pyridine ring of **1c** at a distance of 2.35 Å and 4.39 Å, respectively. His60 and Val218 had established hydrogen bonds with the oxygen of the nitro group at 2.63 Å and 2.68 Å apart, respectively. The chlorine of the phenyl ring formed one hydrophobic alkyl interaction at a distance of 3.91 Å with Met61 and two hydrophobic pi-alkyl connections at distances of 4.61 Å and 4.51 Å with His60 and Phe197, respectively. 3NM8 protein contains residues Phe197 and Pro201 which were assumed to have hydrophobic interactions with the delocalized electron of the pyridine ring. Amino acid residue His208 produced a hydrophobic pi–pi interaction with the delocalized electron of the phenyl ring. When compound **1c** was docked against 2R7A, it exhibited better binding affinity score (−8.9 kcal mol^−1^) than **1c**–3NM8 which justified the improved interactions with *S. dysentery*. From the nonbonding interaction profile of the **1c**–2R7A complex, Trp68 residue of protein and carbonyl oxygen united by a hydrogen bond from a distance of 2.28 Å. Another residue, Arg111, initiated an electrostatic interaction with the pi-electrons of the pyridine ring at a distance of 3.74 Å where Ser32 was assumed to have a hydrogen bond with the nitro group from 2.66 Å. Three hydrophobic interactions were observed by Leu257 with the chloride group (4.49 Å), phenyl ring (5.11 Å) and the pyridine ring (5.06 Å), respectively. Delocalized electrons of the phenyl ring had a hydrophobic pi-sigma bond with Thr52 at 2.76 Å. When compound **1c** was docked against 5JBO, it exhibited the highest binding affinity value of −9.0 kcal mol^−1^. In this complex, Asn241 interacted with the carbonyl oxygen at a distance of 1.93 Å to form a hydrogen bond. Gln319 and Asp243 displayed hydrogen bonds with the nitro group and hydroxyl group at distances of 2.70 Å and 2.42 Å, respectively. The pyridine ring of **1c** was involved in two hydrogen bonds with Glu384 and Glu172 at distances of 3.22 Å and 2.46 Å. Additionally, an electrostatic bond was observed with Glu441 and three hydrophobic bonds with Trp434, Phe450, and Tyr316 residues. A pi-alkyl interaction was displayed between the chloride substituent of the phenyl ring and Trp357 at 3.73 Å. Based on the binding affinity, most of the compounds revealed maximum affinity against 5JBO co-crystal of *T. harzianum*, ranging from −8.2 to −9.0 kcal mol^−1^ which is presented in table 6.

**Table 6.** The binding affinity of the synthesized derivatives against target receptor proteins.

compound	binding affinity (kcal mol^−1^) against target receptor proteins		
	3NM8	2R7A	5JBO
**1a**	−6.6	−8.2	−8.9
**1b**	−6.8	−6.9	−8.9
**1c**	−7.4	−8.9	−9.0
**1d**	−7.0	−8	−8.2
**1e**	−6.8	−7.4	−8.4
**1f**	−6.9	−7.3	−8.6
**1g**	−6.3	−7.4	−8.5

### 3.4. Molecular dynamics simulation

**Figure 6.**
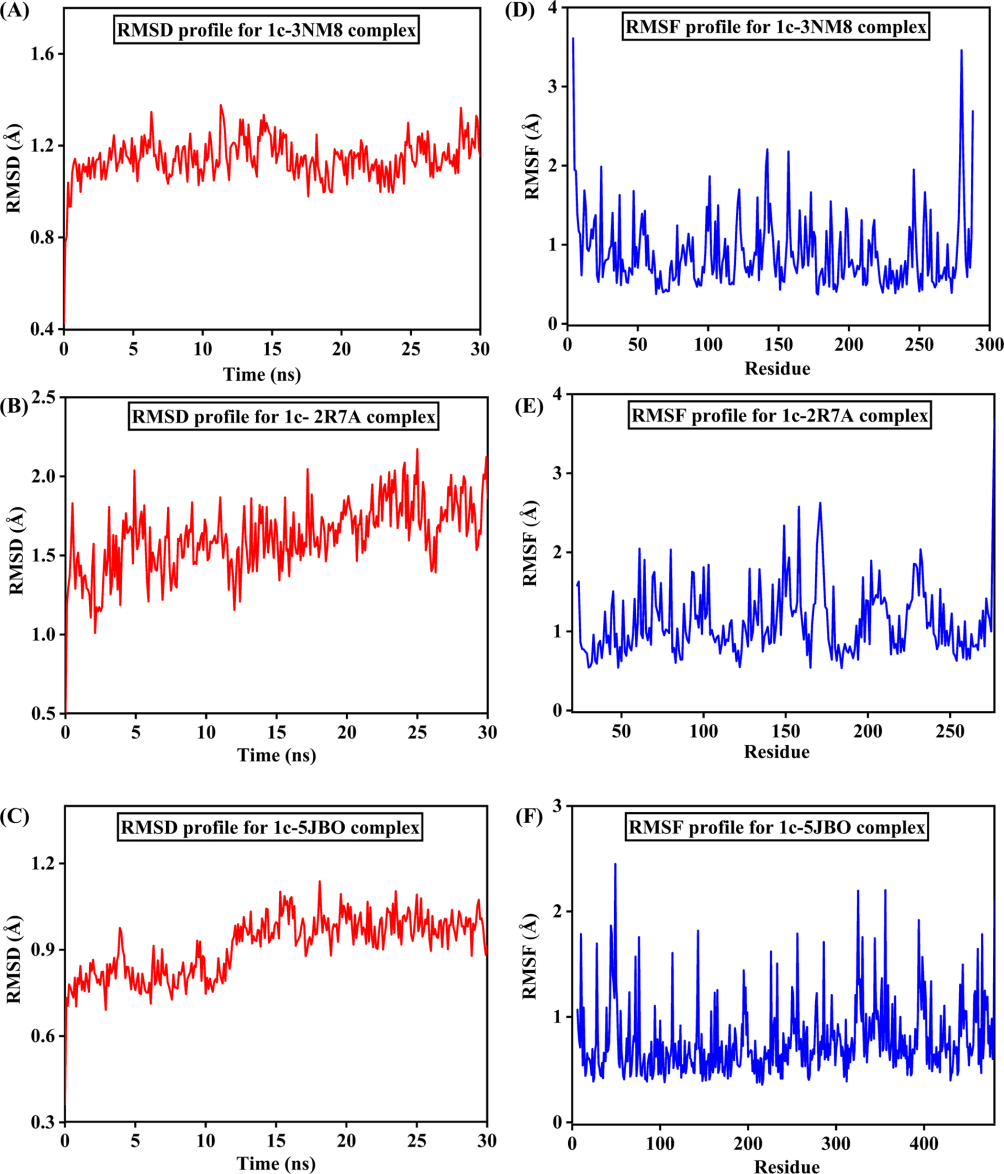
The RMSD and the RMSF plots for the three protein–ligand systems with respect to equilibrated initial structure. (*a*) RMSD plot of **1c**–3NM8 complex, (*b*) RMSD plot of **1c**–2R7A complex, (*c*) RMSD plot of **1c**–5JBO complex, (*d*) RMSF plot of **1c**–3NM8 complex, (*e*) RMSF plot of **1c**–2R7A complex and (*f*) RMSF plot of **1c**–5JBO complex.

**Figure 7.**
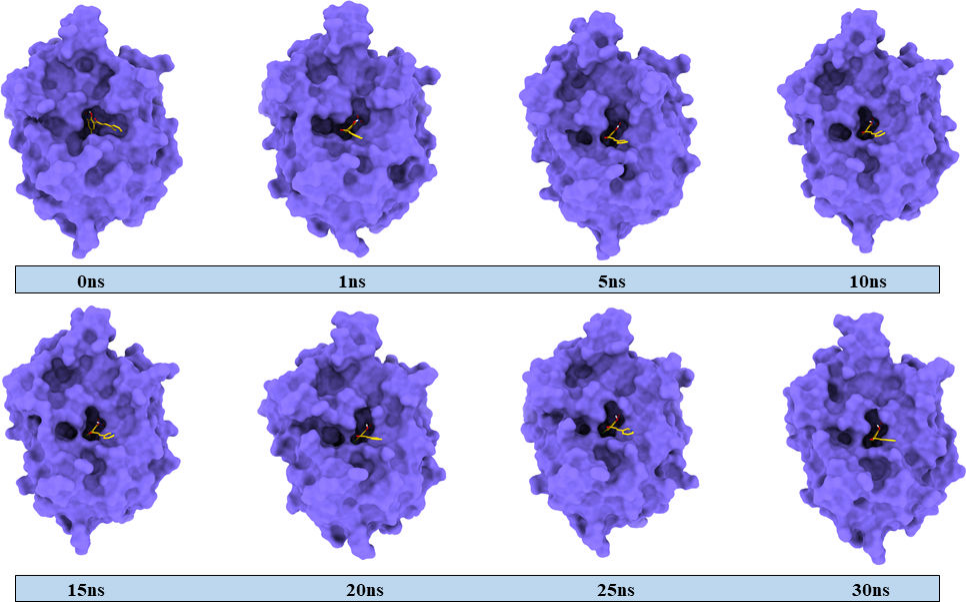
MD-simulated snapshots of complex **1c**–3NM8 during 30 ns trajectory time.

**Figure 8.**
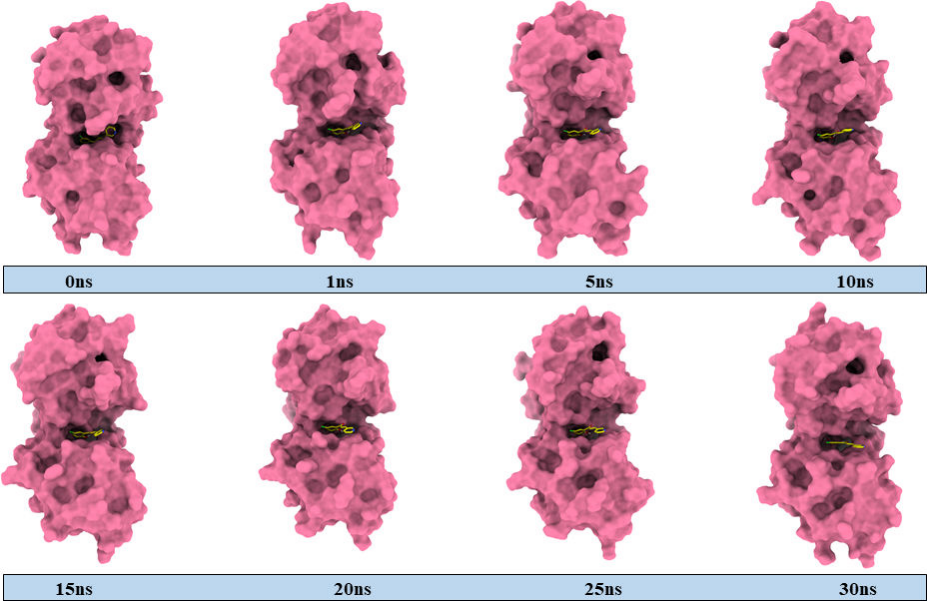
MD-simulated snapshots of complex **1c**–2R7A during 30 ns trajectory time.

**Figure 9.**
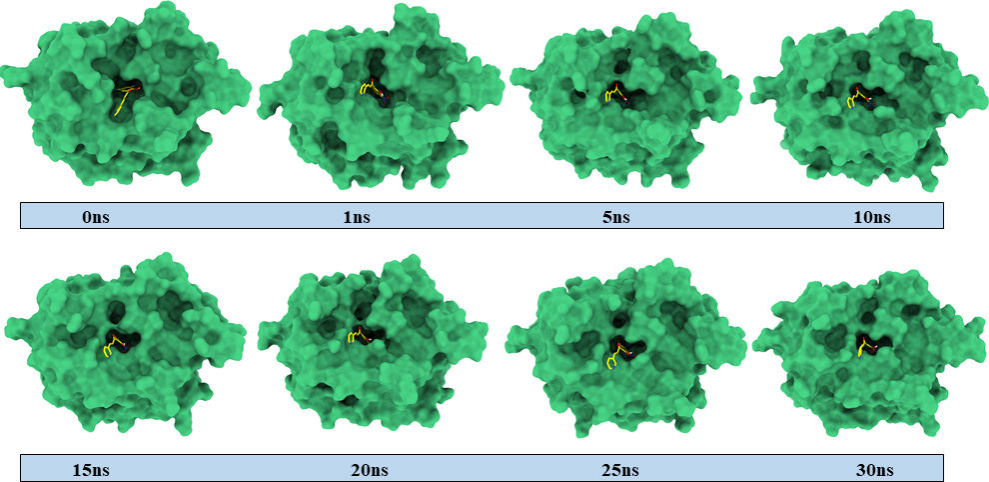
MD-simulated snapshots of complex **1c**–5JBO during 30 ns trajectory time.

In several areas of molecular modelling, MD simulation is frequently employed as a potent drug design technique. In the context of docking, MD simulation is considered as one of the most effective methods to demonstrate the stability and flexibility of both ligand and protein. Simulation of the docked complexes **1c**−3NM8, **1c**−2R7A and **1c**−5JBO was run for 30 ns, and the results were scrutinized using RMSD and RMSF of the protein, as presented in figure 6. The RMSD of alpha carbon atoms for all systems of the complexes was assessed to predict their stability. It is observed from figure 6*a* that the **1c**−3NM8 complex exhibits a stable RMSD plot. Throughout the whole simulation period, no significant fluctuations occurred in either an upward or downward direction. The maximum RMSD value of 1.38 Å was observed at 11.3 ns while the average RMSD was found at 1.15 Å during the whole trajectory. The RMSD plot for the **1c**−2R7A complex is a stable RMSD with an average of 1.62 Å, depicted in figure 6*b*. The RMSD profile of **1c**−5JBO complex with an average of 0.92 exhibited a decent rise of RMSD from 0.74 to 1.03 Å at 10.5 to 13.2 ns (figure 6*c*). While evaluating the RMSD profile along with respective MD snapshots (figures 7–9), it was revealed that in all three complexes, ligand **1c** remains stable in its binding site without any conformational changes [44].

RMSF mainly reveals the system’s elasticity for each amino acid residue that indicates the structural variation within the protein. Residues having RMSF values below 2.5 Å are identified as flexible zones for strong interactions [45]. A high RMSF value reflects greater displacement of the compound from its binding sites. Figure 6*d* exhibits the RMSF plot of the **1c**–3NM8 complex where residues such as Lys4, Gln142, Lys157, Arg280 and Ile288 were assumed to be flexible. This complex presented with most of the protein residues being in minimal range which was within 2.00 Å and the average RMSF was 0.89 Å. From the RMSF of **1c**–2R7A (figure 6*e*) it was found that compound **1c** promoted local flexibility at Lys61, Arg80, Arg149, Lys158, Gly171, Arg232 and Gln277; the remaining residues have RMSF values below 2. The RMSF of the **1c**–5JBO complex displayed a flexible range for residues like Asn49, Glu325, Phe356 and Arg480. All the amino acid residues were found to have RMSF values lower than 2.50 Å, with the average being 0.77 Å (figure 6*f*).

## 4. Conclusion

In this study, we synthesized two series of new chalcone derivatives incorporating pyridine and thiophene moieties. The structures were characterized via spectroscopic data including IR, ^1^H NMR and HRMS. Antimicrobial susceptibilities were examined where compound **1c** produced some excellent zones of inhibition against most of the microbial discs. It exhibited higher activity against all fungal strains compared to the standard and some topmost activities against *B. cereus* (22.3 ± 0.6 mm), *S. sonnei* (43.3 ± 0.6 mm) and *S. boydii* (34.0 ± 1.0 mm) compared to the standard ceftriaxone (20.3 ± 0.6 mm, 40.3 ± 0.6 mm and 25.7 ± 0.6 mm). ADME properties of the analogues were in favourable range for all compounds and displayed low adverse effects. Compound **1e** showed maximum drug-likeness and drug score. The computed ADMET data demonstrate that thiophene-containing compounds revealed higher drug score compared with pyridine-bearing analogues. Molecular docking was performed against three protein co-crystals for all six derivatives while three protein–ligand complexes of compound **1c** were run for simulations owing to its good wet laboratory activity. Each of the complexes displayed stable interaction during the 30 ns of trajectory and the analysed data indicated minimal conformational changes of the composites. All given data demonstrated could promote some engrossing insight towards and will play a pivotal role in drug discovery.

## Data Availability

All data are available in the main text or the electronic supplementary material [46].
